# An exploratory study on the efficacy and safety of a BCAA preparation used in combination with cardiac rehabilitation for patients with chronic heart failure

**DOI:** 10.1186/s12872-017-0639-6

**Published:** 2017-07-27

**Authors:** Munenori Takata, Eisuke Amiya, Masafumi Watanabe, Yumiko Hosoya, Atsuko Nakayama, Takayuki Fujiwara, Masanobu Taya, Gaku Oguri, Kanako Hyodo, Naoko Takayama, Nami Takano, Tomoe Mashiko, Yukari Uemura, Issei Komuro

**Affiliations:** 1 Department of Cardiovascular Medicine, Graduate School of Medicine, The University of Tokyo, 7-3-1 Hongo, Bunkyo-ku, Tokyo, 113-8655 Japan; 2 Clinical Research Support Center, The University of Tokyo Hospital, Tokyo, Japan; 3 Nursing Department, The University of Tokyo Hospital, Tokyo, Japan; 4 Department of Rehabilitation, The University of Tokyo Hospital, Tokyo, Japan; 5 Suxac Inc., Tokyo, Japan

## Abstract

**Background:**

Sarcopenia is generally complicated with patients with chronic heart failure (CHF) and its presence negatively affects the course of heart failure, however effective nutritional intervention had not been elucidated yet. The primary objective of this study is to explore whether the addition of a branched-chain amino acid (BCAA) preparation for cardiac rehabilitation (CR) of patients with CHF further improves cardiopulmonary functions, skeletal muscle functions, and metabolism in comparison with conventional CR.

**Methods:**

This is a randomized, parallel-group comparative study. The elderly patients that were participated in CR and complicated with left ventricular systolic or diastolic dysfunction are randomized into two groups, CR + BCAA and CR. 20 weeks later, the second randomization is performed, which divide subjects into two groups with and without BCAA intervention without CR. Primary outcome measure is the rate of change of the anaerobic threshold workload from baseline to post-intervention. Secondary outcome include parameters of exercise capacity, cardiac function and psychological status.

**Discussion:**

In the current study the effect of a promising new intervention, BCAA, will be assessed to determine whether its addition to CR improve exercise capacity in patients with heart failure, who are generally complicated with sarcopenia.

**Trial registration:**

This clinical trial was registered with the University Hospital Medical Information Network—Clinical Trials Registry (UMIN–CTR; JPRN–UMIN R000022440).

## Background

Shortness of breath and labored breathing occur during daily activities in patients with chronic heart failure (CHF). These symptoms are caused by decreased systolic and diastolic cardiac function. Decreased exercise tolerance because of heart failure is also likely to be complicated with skeletal muscle weakness [1]. Indeed, the exercise tolerance of patients with CHF is known to be strongly related to skeletal muscle strength and muscle mass [2]. A decrease in skeletal muscle strength and muscle mass (sarcopenia) is an important factor that largely affects the subjective symptoms of patients with CHF. Furthermore, sarcopenia is generally complicated with several abnormalities such as metabolic disorders and decreased vascular function [3]. Sarcopenia is often induced by physical deconditioning because of rest during CHF treatment, and it can lead to a condition known as cachexia if concurrent metabolic disorders exist. Approximately 20% CHF patients are believed to suffer from sarcopenia, whereas 10% suffer from cachexia [4], and both are generally observed in elderly patients with cardiovascular disease [5]. Japan is experiencing an increasingly aging population; thus, CHF with coexisting sarcopenia and cachexia have become a big burden.

From the fact that 10%–50% of CHF patients who undergo a single pharmacotherapy intervention are repeatedly admitted for heart failure within a period of 3–6 months, it is evident that the presently available CHF treatments are quite insufficient. With a focus on sarcopenia and cachexia, cardiac rehabilitation is another comprehensive approach that improves cardiopulmonary functions, leading to the suppression heart failure events. Furthermore, in addition to an increase in exercise tolerance, cardiac rehabilitation has been shown to improve left ventricular function, endothelial function, and sympathetic activity, which may have favorable effects on the course of heart failure [6–10].

Branched-chain amino acid (BCAA) preparations have been available for purchase in the market since 1996 with the aim of correcting amino acid imbalance in the blood in patients with decompensated cirrhosis and improving hypoalbuminemia. BCAAs play an important role in the formation of skeletal muscles because they account for approximately 35% of the essential amino acids that form these skeletal muscles [11]. Indeed, BCAA preparations have been reported to promote postoperative wound healing and recovery from muscle fatigue after exercise and enhance muscle strength [12]. Its easily absorbable trait may also fit the treatment of heart failure, because patients with CHF often decreased digestive abilities because of cachexia. Furthermore, reports from animal experiments have revealed that BCAA had some positive effects on heart failure through the improvement on the function of the skeletal muscle mitochondria [13, 14]. Therefore, incorporation of BCAA in cardiac rehabilitation is expected to have additional effect on the improvement of cardiopulmonary and skeletal muscle functions. In this study, we hypothesized that the concurrent use of a BCAA preparation during cardiac rehabilitation has synergistic effects on the clinical course of CHF as well as improvement of skeletal muscle function, and metabolism. In addition, we would also like to investigate whether continued administration of the BCAA preparation after cardiac rehabilitation preserves cardiopulmonary function and skeletal muscles.

### Aim

The primary objective of this study is to explore whether the addition of a BCAA preparation for cardiac rehabilitation of patients with CHF further improves cardiopulmonary functions, skeletal muscle functions, and metabolism in comparison with conventional cardiac rehabilitation. In addition, we will also explore whether continued administration of a BCAA preparation after cardiac rehabilitation can preserve cardiopulmonary and skeletal muscles functions.

### Methods/Design

#### Patient enrollment

Patients with CHF was evaluated in the University of Tokyo Hospital and recruited if they fulfilled the following criteria: (1) Participation in a monitored cardiac rehabilitation program for CHF, (2) age ≥ 65 years, (3) experiencing left ventricular systolic dysfunction [ejection fraction (EF) <40% on echocardiography] or diastolic dysfunction (EF > 40% and E/e′ >15).

#### Exclusion criteria

A patient who meets any of the following criteria will not be included in the study: (1) presence of a congenital BCAA metabolism disorder, (2) presence of contraindications to cardiac rehabilitation, (3) pregnant or breast-feeding patient, (4) presence of other conditions, which make the patient unsuitable for the study according to the judgment of the principal investigator (PI) or coinvestigator (Co-I).

### Study methods

**Fig. 1:**
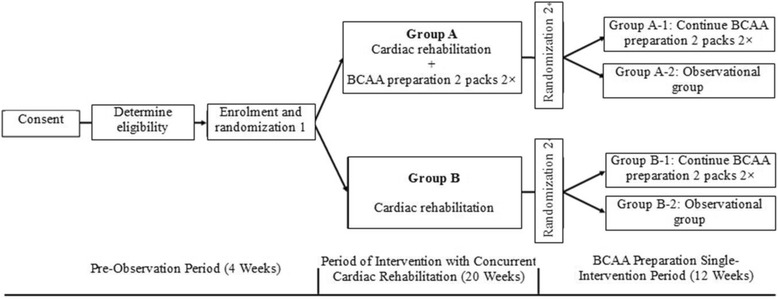
Schematic of study design

This is a randomized, active-controlled, parallel-group comparative study. Study outline is presented in Fig. 1. The study consists of three periods—pre-observation, intervention with concurrent cardiac rehabilitation, and BCAA single intervention. In pre-observation period (Day 1 to Week 4), the consent of patients undergoing cardiac rehabilitation at the hospital for CHF will be obtained, and their eligibility will be determined by blood tests and imaging tests (echocardiogram). In the period of intervention with concurrent cardiac rehabilitation (for 18–22 weeks), patients who are confirmed eligible for study enrollment will be randomized into either group A (CR + BCAA) or group B (CR). BCAA is orally supplemented with one pack of BCAAs granules (Aminovact Combination Granules ®, Nichi-Iko Pharmaceutical Co, Toyama, Japan) which includes 1144 mg of L-valine, 1904 mg of L-leucine and 952 mg of L-isoleucine, two times a day. Cardiopulmonary exercise test (CPX) will be performed on each subject in both groups before intervention with the BCAA preparation as well as at Week 14 and Week 20 of the intervention with cardiac rehabilitation. In the BCAA preparation single intervention period (20–24 weeks), re-randomization, i.e., randomization 2, will be performed after the cardiac rehabilitation program has ended. Subjects in group A will be assigned to Group A-1 or A-2, whereas those in Group B will be assigned to Group B-1 or B-2. As described in Fig. 1, subjects assigned to Group A-1 will continue the BCAA preparation. Similarly, subjects in Group B-1 will start taking the BCAA preparation. The BCAA preparation will be discontinued for subjects assigned to Group A-2, and subjects in Group B-2 will not undergo intervention with the BCAA preparation throughout the study period.

This clinical study is in accordance with International Conference on Harmonization of technical requirements for registration of pharmaceuticals for human use (ICH-GCP). Furthermore, the clinical study is conducted with strict observance of ethical guidelines and the latest Declaration of Helsinki. This clinical trial was registered with the University Hospital Medical Information Network—Clinical Trials Registry (UMIN–CTR; JPRN–UMIN R000022440). Also, the protocol was approved by the institutional review board of Tokyo University Hospital.

### Enrollment and assignment

PI or Co-I will obtain the written consent of subjects. The enrollment of subjects who have given their consent and the data of all subjects will be entered into the electronic data capture (EDC) system (Viedoc, PCG Solutions, Uppsala, Sweden). For randomization 1, the subjects will be stratified into two groups (groups A and B as mentioned earlier) according to (1) age (>70 or ≤70 years) and (2) anaerobic threshold (AT) workload (≥30 watts or <30 watts) in the first CPX performed at the time of consent-taking. Randomization 2 will be performed after the intervention period with concurrent cardiac rehabilitation has ended. The stratification factors are (1) the age of the subject (>70 or ≤70 years) and (2) the rate of change in the AT workload in CPX after intervention with concurrent cardiac rehabilitation has ended (≥25% or <25% for group A and ≥15% or <15% for group B).

### Outcome measures

Primary outcome measure is the rate of change of the AT workload from baseline to post-intervention with concurrent cardiac rehabilitation. Secondary outcome measures include the following: (1) The rate of change from baseline which is performed in the pre-observation period or reference day [Peak VO_2,_ left ventricular EF in the echocardiogram, total points of the Hospital Anxiety and Depression Scale (HADS), muscle strength (grip strength and lower-limb extension power), absolute values of interleukin-6 (IL-6), and tumor necrosis factor (TNF)-α], Time taken for 50% subjects to achieve a 15% improvement in AT workload, which is defined as an event. (2) The rate of change from post-intervention period with cardiac rehabilitation (20 weeks later) to post-single intervention with the BCAA preparation (32 weeks later) [Peak VO_2_, AT workload, total points of HADS, muscle strength (grip strength and lower-limb extension power))], (3) The frequency of serious adverse events (SAEs) throughout the study period.

### Observations and examinations

After enrollment, PI or Co-I collect all data prospectively whenever feasible. Data include the following: (1) the patient’s background: gender, date of birth, height, body weight, complications, past medical history, etiology of heart failure, coronary risk factors (hypertension, dyslipidemia, diabetes, hyperuricemia, and age > 45 years), and history of present illnesses; (2) compliance to IP administration (records of the day on which the BCAA preparation was first prescribed and the day of the last prescription) and the number of visits to the hospital for cardiac rehabilitation (per week); (3) concomitant medications; (4) subjective symptoms (Borg scale during cardiac rehabilitation) and objective findings; (5) adverse events (AE)^a^: details of AE, date and time of AE onset and resolution, severity, treatments, outcome, assessment of severity, and causal relationship with IP will be entered into the medical records and EDC. Follow-up investigations will be performed, if necessary. The levels of severity are defined as follows: (1) mild: treatment is not required and IP administration can continue, (2) moderate: IP administration can continue with some treatment, and (3) severe: IP administration will be or should be discontinued.

Characteristic patient data includes following parameters (Table 1): (1) Vital signs: Blood pressure before the start of cardiac rehabilitation (before warm-up exercise), pulse rate (in sitting position), as well as blood pressure and pulse rate at the time of outpatient visits (in sitting position); (2) CPX^b^: AT workload, VO_2_ AT, peak VO_2_, and ΔVO_2_/Δ work rate (WR); (3) Complete blood count (CBC), biochemistry tests and blood plasma tests^c^: White blood cell, red blood cell, hemoglobin, hematocrit, platelet, total protein, albumin, cholinesterase, aspartate aminotransferase, alanine aminotransaminase, gamma-glutamyl transpeptidase, blood urea nitrogen, creatinine, C-reactive protein, and brain natriuretic peptide; (4) Cytokine blood tests^d^: TNF-α and IL-6; (5) Echocardiography^e^: left ventricular diastolic diameter (M mode), left ventricular EF (M mode), E/A, E/e’, and left atrial diameter; (6) Emotional outcome measure^f^: Total points of HADS; (7) Assessment of muscle strength^g^: Grip strength and lower-limb extension power; (8) Events of hospitalization because of heart failure during the study period.

**Table 1:**
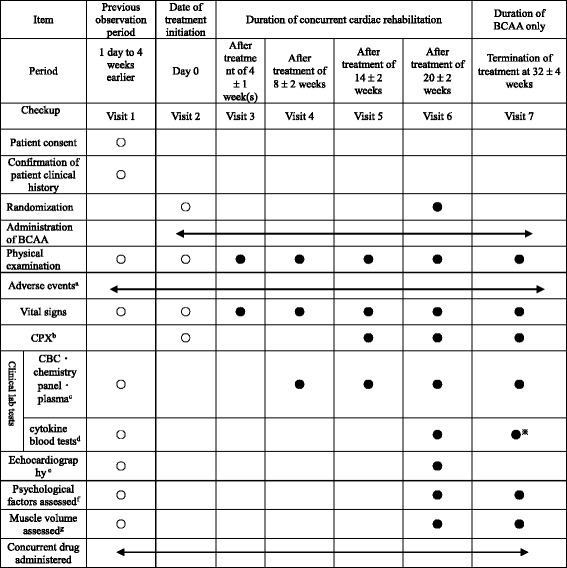
Study schedule

○ Indicates items that occur before administration of BCAA

● Indicates items that occur after administration of BCAA

※ Indicates items that occur after suspension of the study

### Statistical considerations

The sample size was calculated according to the estimation as follows. We hypothesized that following 6 months of cardiac rehabilitation, there will be an AT workload improvement of 25% (SD: 10%). With the addition of BCAA, we hypothesized that there will be an AT workload improvement of 25% (SD: 10%). Setting the two-sided significance level at5% and power at 90%, the required sample size was 43 cases. Assuming a 5% rate of subjects leaving the study, we chose 48 cases as the target number.

According to the study results, differences (%) in primary outcome between each outcome measurement between A and B groups will be evaluated using the t-test. With respect to differences in outcome measurement (%) in secondary endpoint between groups A and B, the t-test will be used to analyze statistical significance. Similarly, the difference between B-1 and B-2 sub-groups will be analyzed using the t-test. Also,, after confirming the assumption that there are no major differences among the treatment groups (no interaction), the two-way ANOVA will be used to test the treatment effect for addition of a BCAA, and maintenance effect for continuing administration of a BCAA by including group A/B and +1/+2 as factors. A box-plot will be used to visualize primary outcome and secondary outcomes between the 4 groups (A + 1, A + 2, B + 1, B + 2). Finally, defining an AT workload improvement of 15% as an event, a Kaplan–Meier curve will be plotted to compare the curves between groups A and B, and estimate the occurrence of an event rate of 50% of each group.

## Discussion

Cardiac rehabilitation includes nutritional interventions in addition to exercise training [15]. The main aim of nutritional intervention seems to be an assist of effect derived from exercise training interventions; however, the nutritional modification might itself have favorable effects on the skeletal muscle or cardiopulmonary capacity leading to the improvement of exercise capacity in patients with heart failure [16]. The study focused on the elderly patients with heart failure, who are potentially complicated with sarcopenia. However, we did not limit the study patients by excluding patients without subjectively confirmed sarcopenia because we would investigate the effectiveness of more general and easy use of BCAA agents for cardiovascular patients.

One unique point in this study is that there are two terms of nutritional interventions, one with exercise training and one without it. The results help distinguishing the additional effect on exercise training or original effect derived from the nutritional intervention itself. In addition, the two-time randomization method may decrease the influence of the result of first randomization on the intension to exercise training.

In the current study the effect of a promising new intervention will be assessed to determine whether BCAA addition improve exercise capacity in patients with heart failure, who are generally complicated with sarcopenia. We hope this study, with its design and data collection, will present novel insights addressing the effective nutritional intervention in cardiac rehabilitation.

## Abbreviations

AT: Anaerobic threshold
BCAA: Branched-chain amino acid
CBC: Complete blood count
CHF: Chronic heart failure
Co-I: Coinvestigator
CPX: Cardiopulmonary exercise test
EDC: Electronic data capture
EF: Ejection fraction
HADS: Hospital anxiety and depression scale
ICH-GCP: International Conference on Harmonization of technical requirements for registration of pharmaceuticals for human use
IL-6: Interleukin-6
PI: Principal investigator
SAEs: Serious adverse events
TNF: Tumor necrosis factor
